# Experience of living with multimorbidity and health workers perspectives on the organization of health services for people living with multiple chronic conditions in Bahir Dar, northwest Ethiopia: a qualitative study

**DOI:** 10.1186/s12913-023-09250-9

**Published:** 2023-03-09

**Authors:** Fantu Abebe Eyowas, Marguerite Schneider, Shitaye Alemu, Fentie Ambaw Getahun

**Affiliations:** 1 School of Public Health, College of Medicine and Health Sciences, Bahir Dar University, Bahir Dar, Ethiopia; 2 Department of Psychiatry and Mental Health University of Cape Town, Alan J Flisher Centre for Public Mental Health, Cape Town, South Africa; 3 School of Medicine, College of Medicine and Health Sciences, University of Gondar, Gondar, Ethiopia

**Keywords:** Multimorbidity, Health system, Qualitative methods, Thematic analysis, Ethiopia

## Abstract

**Background:**

Multimorbidity-the simultaneous occurrence of two or more chronic Non-Communicable Diseases) in an individual is increasing globally and challenging health systems. Although individuals living with multimorbidity face a range of adverse consequences and difficulty in getting optimal health care, the evidence base in understanding the burden and capacity of the health system in managing multimorbidity is sparse in low-and middle-income countries (LMICs). This study aimed at understanding the lived experiences of patients with multimorbidity and perspective of service providers on multimorbidity and its care provision, and perceived capacity of the health system for managing multimorbidity in Bahir Dar City, northwest Ethiopia.

**Methods:**

A facility-based phenomenological study design was conducted in three public and three private health facilities rendering chronic outpatient Non-Communicable Diseases (NCDs) care in Bahir Dar City, Ethiopia. Nineteen patient participants with two or more chronic NCDs and nine health care providers (six medical doctors and three nurses) were purposively selected and interviewed using semi-structured in-depth interview guides. Data were collected by trained researchers. Interviews were audio-recorded using digital recorders, stored and transferred to computers, transcribed verbatim by the data collectors, translated into English and then imported into NVivo V.12 software for data analysis. We employed a six-step inductive thematic framework analysis approach to construct meaning and interpret experiences and perceptions of individual patients and service providers. Codes were identified and categorized into sub-themes, organizing themes and main themes iteratively to identify similarities and differences across themes, and to interpret them accordingly.

**Results:**

A total of 19 patient participants (5 Females) and nine health workers (2 females) responded to the interviews. Participants’ age ranged from 39 to 79 years for patients and 30 to 50 years for health professionals. About half (*n* = 9) of the participants had three or more chronic conditions.

The key themes produced were feeling dependency, social rejection, psychological distress, poor medication adherence and poor quality of care.

Living with multimorbidity poses a huge burden on the physical, psychological, social and sexual health of patients. In addition, patients with multimorbidity are facing financial hardship to access optimal multimorbidity care. On the other hand, the health system is not appropriately prepared to provide integrated, person-centered and coordinated care for people living with multiple chronic conditions.

**Conclusion and recommendations:**

Living with multimorbidity poses huge impact on physical, psychological, social and sexual health of patients. Patients seeking multimorbidity care are facing challenges to access care attributable to either financial constraints or the lack of integrated, respectful and compassionate health care. It is recommended that the health system must understand and respond to the complex care needs of the patients with multimorbidity.

**Supplementary Information:**

The online version contains supplementary material available at 10.1186/s12913-023-09250-9.

## Introduction

Multimorbidity, the simultaneous occurrence of two or more chronic non-communicable diseases (NCDs) in an individual, is an emerging global public health problem [1].

Although studies are diverse in methodology and context, the prevalence of multimorbidity is increasing [2]. Recent reviews reported a pooled prevalence of 42.4% in high-income countries (HICs) [3], 43% in Latin America and Caribbean [4] and 36.4% in low-and middle-income countries (LMICs) [5]. A scoping review of multimorbidity studies in LMICs reported wide prevalence estimates ranging between 3.2 to 90.5% [6], with a recent facility based study among adults attending chronic outpatient NCDs care in northwest Ethiopia showing a prevalence of 54.8% [7].

The prevalence of multimorbidity increases with age [5, 6], economic deprivation [5, 8], female gender [5, 9], obesity [5, 10, 11] and among individuals with limited social network and support systems [7, 12]. Although the prevalence of multimorbidity is highest among adults aged 65 or older, younger persons also represent a large proportion of those with multimorbidity [13]. In addition, multimorbidity appears 10–15 years earlier in people living in the most deprived areas than for those living in the most affluent areas [8].

Individuals living with multiple chronic conditions face a range of adverse consequences, including premature mortality [14], poor quality of life [15, 16], impaired functioning [5, 17], treatment burden [18], reduced productivity [19] and high cost of care [20, 21], among others. In the era of high vulnerability to life-threatening infections such as COVID-19, the probability of dying prematurely is also greater among patients with multimorbidity [22, 23].

Management of people with multimorbidity is challenging in many ways [24]. On the one hand, there is no conclusive evidence on the best model of care [24–26]. On the other hand, the current models of care tend to focus on diseases in isolation rather than the needs and circumstances of the person with complex care needs as a whole [27–29].

In addition, the incurable nature of the diseases requires patients to have regular investigations, take different medications and attend multiple medical care follow ups and adhere to lifestyle recommendations [30–32], all of which pose psychological and financial burden [33]. Moreover, caring for people with multiple chronic health conditions is challenging because there are several potentially competing treatments and health outcomes [24].

In general, although patients with multimorbidity require a holistic approach, clinicians may not have the key skills needed to balance the priorities given to single-disease and management of multiple long-term conditions [32]. The lack of physicians’ time further limits the provision of optimal care and efforts to meaningfully engage patients in collaborative decision-making about their care [24].

The lack of patient-centered care, fragmented approach and poor coordination lead patients to see multiple health professionals in primary and secondary care facilities [34], resulting in these patients being dissatisfied and sometimes confused with the care they receive [21]. Even if treatment is appropriate, inadequate use of medication and polypharmacy may increase the risk of complications [35]. This is particularly common in low-income countries such as Ethiopia, where access to the diagnosis and management of chronic conditions is inadequate [36, 37].

Health services in Ethiopia are organized around single conditions, although patients could have more than one diagnosed condition. Further, clinicians tend to use one-size-fits-all chronic care guidelines in a fragmented and siloed fashion [38]. Visits to different specialists often in different facilities, suggests that people living with multiple conditions are receiving potentially uncoordinated care that may lead to negative health outcomes, including mortality.

Moreover, multimorbidity is relatively new in the health system and health professional education context in the country, and there are substantial gaps in our knowledge about people’s lived experiences of multimorbidity. A better understanding of the journey and challenges patients are facing while seeking care and exploring the views and perspectives of care providers on the organization and availability of essential resources to care for and improve the outcomes of people with multimorbidity is a priority research agenda [30, 39].

This study aimed at understanding the lived experiences of patients with multimorbidity and perspective of service providers on multimorbidity and its care provision, and capacity of the health system for managing multimorbidity in Bahir Dar City, northwest Ethiopia.

## Methodology

This study is part of an ongoing research project whose protocol has been published elsewhere [40].

### Design

A phenomenological study design was employed to explore participants’ experience of living with multimorbidity and perceptions of service providers on the concept of multimorbidity, their experience in managing multimorbidity and their views on the health system’s capacity and its effectiveness in responding to the needs of people seeking chronic multimorbidity care. The phenomenological study design is suitable when a researcher is interested to deeply understand about the views, perceptions, perspectives and experiences of study subjects on the phenomenon under study [41].

### Study setting

This is a facility-based qualitative study conducted in public and private health facilities rendering health services in Bahir Dar city, Ethiopia. The city is the capital of the Amhara regional state, the second largest and populous region in the country with a population of ~ 30 million people.

### Study population, sample and recruitment of participants

A broad sample of health facilities, patients and health workers was purposively recruited to capture maximum variation of the phenomenon under study [42]. Participating health facilities involved three public hospitals (one primary, one tertiary and one specialized teaching hospitals), one private general hospital and two private specialized clinics that provide long-term NCDs care.

Nineteen patient participants with two or more chronic NCDs were purposively selected with the aim to satisfy maximum variation sampling method based on the nature of chronic conditions the patients are living with, sex, age and residence (Table 1). For the service providers, six medical doctors (2 GPs, 1 internist, 1 cardiologist, 1 endocrinologist and 1 internal medicine resident) and three nurses working in chronic outpatient care departments were recruited. The two sub-specialists (cardiologist and endocrinologist) and one nurse were working in both public and private health facilities, while the rest were working in public hospitals only.

**Table 1:** Characteristics of patient participants Bahir Dar, Ethiopia

Sex	Age	Facility	Number and types of conditions	Duration of living with the disease/s (years)
Female	50	Public specialized teaching hospital	3 (HPN, KD, Gastritis)	13
Female	75	Public specialized teaching hospital	3(HPN, heart problem and RA)	16
Male	50	Public specialized hospital	2(DM, HPN)	6
Female	55	Public specialized hospital	3(HPN, DM and hypercholesteremia)	7
Male	79	Public specialized hospital	3(HPN,DM &KD)	30
Male	74	Public specialized hospital	3(HPN, DM and hypercholesteremia)	17
Female	39	Public specialized teaching hospital	3(HPN, HD, TB)	2.5
Female	50	Public specialized teaching hospital	4(HPN, HF, RA, Asthma	2
Male	66	Public specialized teaching hospital	3(DM,HPN, HD)	15
Male	52	Public primary level hospital	2(HPN, BPH)	8
Male	48	Private general hospital	3(HPN, DM, RF)	24
Female	50	Public primary level hospital	2(HPN, DM)	1
Female	66	Public primary level hospital	2(HPN, DM)	4
Male	45	Private general hospital	2(HPN,DM)	5
Female	54	Private specialized medical center	3(DM, HPN and hypercholestremia)	21
Female	60	Private specialized medical center	2(HPN, DM)	18
Male	75	Private specialized clinic	2(HPN, HD)	7
Male	45	Public specialized hospital	2(HPN, DM)	8
Male	58	Public specialized hospital	2(HPN, DM)	3

Patient participants were recruited on the day of their appointment following the completion of their follow-up care. Medical doctors and nurses who work in the chronic care units of the selected facilities supported the identification and recruitment of study participants. Patients who attended chronic care follow-up for at least six months and care providers who have had at least one-year experience of managing patients with chronic conditions were eligible for the study. All the in-depth interviews were conducted in the vicinity of the facilities where patients attend chronic care follow-up. The first author in collaboration with facility leaders and study facilitators arranged convenient rooms for the interviews in each facility.

### Data collection techniques and tools

#### Patients

Patient participants were interviewed in-person by four PhD fellows and the first author. In-depth interviews were conducted using semi-structured interview guides outlining a broad set of questions crafted and shared with the co-authors for feedback and revision. The topic guide for patients (S1) asked about their views on living with multiple chronic conditions, the impact of multimorbidity on daily living, family management, social functioning and self-management, as well as their experience in accessing health services and their views on care coordination and continuity of care.

The first author organized training and discussion sessions for the data collectors to ensure clarity and shared understanding on the semi-structured tools and the techniques for conducting the interviews. The tool was pilot-tested with two patients in Felegehiwot specialized hospital by the first author and the interviewers together. The authors and data collectors reviewed the process followed in these pilot interviews, listened to the audio recordings, reviewed the field notes, clarified concerns and revised the tool for subsequent interviews.

Data collectors were grouped into two teams, each with two interviewers (one note taker and the other interviewer and audio recorder). Interviews, transcription and coding of the data continued until the first nine patients were interviewed. The first author listened to the recordings and reviewed the transcriptions of the first nine interviews carefully. Gaps noted were perspectives of female patients, Muslims and rural residents and given the importance of such perspectives, the data collectors were advised to recruit patients from these backgrounds as well. The two authors (FAE and FAG) and interviewers held regular meetings to discuss responses to the topic guide and evolving perspectives to improve richness of the remaining data.

Interviews were audio-recorded using digital recorders, stored and transferred to a computer, transcribed verbatim by the data collectors, translated into English and then imported into NVivo V12 software for data analysis. Field notes were taken to complement the general feeling and specific observations made during the interviews.

#### Service providers

The first author conducted in-depth interviews with medical doctors and nurses using a semi-structured tool (S2) to explore the perspectives and experiences of service providers on multimorbidity. Providers were asked about the concept of multimorbidity, the way healthcare is organized and capacity of the health system to screen, diagnose and manage patients with chronic multimorbidity. In addition, the providers were asked about their experiences in managing multimorbidity, challenges in screening and managing multimorbidity and to reflect their opinion about patients’ self-management skills and recommendations to improve access and management of people with multimorbidity. Interviews were audio-recorded using digital recorders, stored and transferred to a computer, transcribed verbatim, translated into English and analyzed using NVivo V12.

#### Quality assurance

The process we followed and experiences of patients and service providers was described to enhance credibility [43]. We have employed maximum variation sampling and clearly described study participants, the study setting and research process to ensure transferability of our findings to a similar context [42–44]. Dependability, the consistency and quality of data collection process, was substantiated with use of field notes and an audit trail of the decisions made during the study [44]. We tried to show confirmability of the interpretations through incorporating participants directs quotes in the findings [43, 44].

#### Data analysis

We employed a six-step inductive thematic analysis approach [45, 46] to construct meaning and interpret experiences and perceptions of individual patients. Thematic analysis is an appropriate and powerful method to analyze a set of experiences, thoughts, or behaviors across a data set [45].

In step 1, the first author listened to all interview recordings while simultaneously reading the transcripts and field notes to understand the overall meaning of responses provided by the participants.

In step 2, each transcript was read line by line to make sense of the data and drive initial coding. The initial codes were organized in MS word to assign coding schemes inductively. Then, focused coding was applied to reduce the volume of the raw information and to identify significant patterns for categorizing and assigning with themes and sub-themes. Codes were identified and categorized into sub-themes and themes iteratively (constant comparison) to compare and identify similarities and differences across themes [47].

**Fig. 1:**
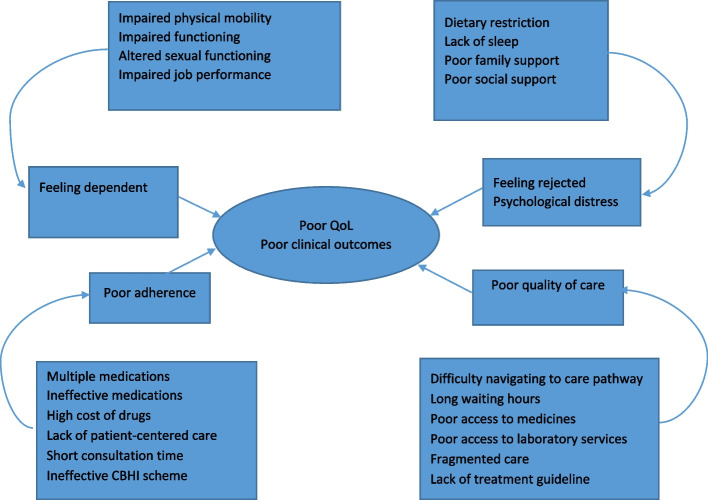
Network of the sub-themes, organizing themes and main themes

Twenty sub-themes (basic themes), five organizing themes and two global themes were constructed (Fig. 1). Sub themes are the most basic premises of characteristics derived from the textual data. The themes that organize the basic themes into a cluster of similar issues are organizing themes. Whereas, global themes encompasses the principal metaphor in the data as a whole. The global themes summarize and make sense of the cluster of lower-order themes abstracted from and supported by the data [46].

In step 5 and 6, we interpreted the themes and developed a written report of the themes generated [43, 45, 46]. The themes constructed are organized around the effect of multimorbidity on physical, psychosocial and sexual health, and functioning, self-management, access to care and organization of health services. Perspectives of health workers in each of the domains studied are described along with the themes related to self-management, access to care, service organization and institutional capacities.

## Results

A total of 19 patient participants responded to the interview. The interview length for patients varied from 17 to 48 min and data saturation was reached after interviewing 16 patients; three more patients were interviewed with no new themes emerging. Percipient ages ranged from 39 to 79 years. About half (9 of the 19 patients) have three or more chronic conditions. All of the patient participants had hypertension as one of the comorbid conditions and diabetes was the second most frequently reported condition (Table 1).

Nine health professionals (6 medical doctors and 3 nurses) were recruited from different facilities to explore their views on multimorbidity and its challenges and to describe how the health system is organized and capacitated to manage multimorbidity in their context. The interview length for health professionals ranged from 26 to 49 min and data saturation was achieved after interviewing five medical doctors and two nurses; one internal medicine resident and one nurse were interviewed with no new themes emerging. Their age ranged between 30 and 50 years with a minimum three years working experience in NCDs management (Table 2).

**Table 2:** Characteristics of service providers enrolled Bahir Dar, Ethiopia

Sex	Age	Profession	Facility	Service year in chronic NCDs care
Male	31	General practitioner	Public specialized hospital	3
Female	37	Nurse professional (BSc.)	Public specialized hospital	4
Male	39	Medical internist	Public specialized hospital	5.5
Male	45	Medical internist + cardiologist	Private specialized clinic + Public specialized teaching hospital	20
Male	50	Medical internist + endocrinologist	Private specialized medical center + Public specialized teaching hospital	25
Male	30	General practitioner	Public specialized hospital	3
Female	38	Post graduate nurse (adult medical nursing)	Public specialized hospital	5
Male	36	Resident physician	Public specialized teaching hospital	7
Male	30	Nurse practitioner	Private specialized clinic	6

### Overview of the thematic analysis findings

About 256 codes were generated (S3). The codes were organized into 21 sub-themes and five organizing themes, including dependency, poor adherence, feeling rejected, psychological distress and poor quality of life (Fig. 1).

The lived experiences of individuals with multimorbidity, service provision and the perspectives of health care providers are presented in accordance with the five organizing themes abstracted above.

#### Feeling dependent

Patients living with multimorbidity face a huge challenge with their physical health. Many patients are in chronic pain and suffer from reduced physical mobility, both of which affect activities of daily living and quality of life (QoL) as reflected in the following narratives:

“I was a soldier and hard worker, but after I had those diseases, I am held back. I stopped working, feeling sad, suffer pain, feel fainting at exertion [shirgata] and unable to go to church. I could have done much at this age, but….you see I am disabled.” [Male, 66, 3 NCDs]

*“The physical limitation is so disabling that I could not take care of myself and go to church. I spend days perhaps weeks at home, gazing around with despair and feeling my diseases progressively worsening.”* [Male, 58, 2 NCDs]

Patient participants reported impaired physical functioning and difficulty in maintaining jobs and accomplishing activities of daily living.

*“I stopped working. I feel pain and discomfort while trying to do the job I used to do. It becomes the responsibility of my husband to earn money to the house. I could not assist him as I did before.”* [Female, 39, 3 NCDs].

*“When it comes to my work it is really hard, I am limited physically and cannot do the work I was doing before, including home based activities.”* [Female, 50, 4 NCDs].

Another patient participant described that he stopped working his fields and restricted to only working in the morning at office. *“I am unable to do the routines. Colleagues help me doing my jobs, including field works. I feel weak and often spend hours sitting. I became less productive, particularly in the last two years.”* [Male, 50, 2 NCDs]

#### Psychological distress

Living with multimorbidity affects patients’ psychological health and social lives because they feel vulnerable and worry about disease management and restrictions while participating in social events.

*“It is difficult to be free from stress and anger. I often feel emotionally unstable, easily annoyed and intolerant to people around me. People do not understand your problem, perhaps they blame it on you and you feel stigmatized and rejected. That is why I prefer to avoid social gatherings.”* [Male, 52, 2 NCDs]

Most patients mentioned that they are suffering from sleep deprivation, and the lack of sleep is posing distress *“I cannot sleep after midnight, although I go to bed late in the evening.”* [Male, 48, 3 NCDs]

Some participants said that multimorbidity affects their sexual life. *“I find it difficult to be sexually active. I have erectile problem and worrying that this may affect my wife.”* [Male, 58, 2 NCDs].

The psychological burden of multimorbidity on the family is well understood by service providers.

*“Living with multiple long-term conditions affects the whole family. Family members also get stressed due to the financial burden they share to cover for laboratories, drugs and follow-up services.”* [Male 45, internist cardiologist]

*“People living with multimorbidity have disrupted quality of life, poor family management and compromised financial security owing to high expense of care, reduced productivity and due to their demand for a full time care taker from their family. This will eventually pose a burden to the family, health system and the country at large.”* [Female, 38 adult medical nursing specialist]

#### Social isolation and rejection

Most patients spoke about being socially isolated when thinking about their conditions and social lives.

*“I tend to separate myself, except in case of funerals. I am now weak, they say please remain at home, ‘simply pray’.”* [Male, 74, 3 NCDs]

Some patients feel that religious leaders do not understand their problems.

*“I often go to holy places to get holy water bath. People there force you to choose one: ‘either the holy water or drugs.’ They do not seem to help you. It is a lip service.”* [Male, 48, 3 NCDs]

Another patient mentioned that he is worried about failing to comply with religious rules*. “I stopped going to church. Because I stopped fasting as I am required to take medicines morning and evening. I live in conflict with my values.”* [Male, 45, 2 NCDs]

However, some patients described feeling supported and trying to establish and maintain strong and supportive relationship with the family and the community they are living with. *“People around me, including my families support me financially and morally to cope with my illnesses.”* [Female, 75, 3 NCDs]

#### Poor adherence to treatment

Adherence to medications and self-management is challenging for patients. Patients spoke of the confusion and stress of taking multiple medications. “*Taking many drugs several times a day for several years is burdensome. I defaulted my regimen several times hoping a herbal medicine [Shiferaw] would help. I also tried sport, but it doesn’t work.”* [Male, 45, 2 NCDs]

Some patients failed to comply with doctors’ advice and pretend they are taking medicines as prescribed.

*“I kept fasting. I lied to doctors that I am taking medications twice a day. Actually, I only take it once per day. I never stopped alcohol and salt intake although doctors recommended that.”* [Female, 55, 3 NCDs]

A doctor who manages patients with chronic illness described the problems of taking multiple medications. “*Individuals living with multimorbidity take several medicines. That will have a biological and psychological effect. There may be drug-to-drug interaction. People may default treatments or take them selectively.”* [Male 31, GP, 31]

Poor adherence is also a commonly held opinion by the nurses too. *“Patients living with multiple chronic conditions face difficulty in adhering to multiple prescriptions and to tolerate medication side effects. They often skip doses and take medications selectively and come with complications later.”* [Female 37, Nurse].

*“Patients take 2–8 tablets per day. Owing to the lack of knowledge, particularly rural residents do not take their treatments according to the prescribed manner. Sometimes, they visit traditional healers to avoid taking drugs. Some of the patients may gradually become fed up [bored with pill burden] taking several medicines for incurable diseases. Some of them default treatments due to financial burden and lack of social support.”* [Male 30, Nurse]

The challenges faced are not only related to medication burden, but also due to prescriptions being frequently changed because first line treatments are being unavailable.

*“I have to take and adapt new therapies every time; because I couldn’t obtain the medicines I am already familiarized and comfortable with.”* [Male, 58, 2NCDs]

A female patient with three morbidities mentioned that the medicines she takes do not work.

*“I have to live until I die. It is Devil’s disease. I am not improving and I told to doctors that the treatment does not work.”* [Female, 54, 4 NCDs]

*“Some patients have doubts about the quality of drugs. I think it is because of the lack of knowledge and we need to work together to clarify that misconception.”* [Male 50, internist endocrinologist]

Conversely, however, some patients learnt to accept the reality and adapted to living with multimorbidity. *“I know that the diseases are dangerous; they could damage me at any point in time. I am taking care of myself, take medicines accordingly, and comply with doctors’ advice. Except for the fear of sudden death and the financial burden, I am okay now.”* [Male, 75, 2 NCDs].

Another patient described his adjustment to living with multimorbidity, as *“I am caring for myself. I optimized my diet and attend medical follow ups; because I know individuals died of [high blood] pressure and sustained a half-body weakness [paralysis] because of a lack of treatment.”* [Male, 52, 2 NCDs]

*“It is my duty to follow doctors’ advice; else I will die. I never skip medicines and appointments. I feel confident that I am able to control the diseases I am living with.”* [Male, 66, 2 NCDs]

Facing high cost of drugs was the other challenge that might have contributed to poor adherence. In this study, almost all of the participants (both patents and service providers) mentioned the cost of medicines and that patients are facing severe financial burden buying drugs from private drug vendors.

*“We spend much of our income to purchase medications; I have to live, no option.”* [Female, 39, 3 NCDs].

*“It is recently that I came to this hospital, because the private hospital I used to attend care was expensive. Unfortunately, medicines are not available here [public hospital]. I am only given the prescriptions to buy drugs outside. That is not helpful, because I cannot afford.”* [Female, 50, 2 NCDs]

The majority of the participants described that they are facing huge financial burden due buying medicines at private pharmacies rather than picking them up at the public facility.

*“Buying medicine in private pharmacies is expensive*. *Medicines are often unavailable in the [public] hospital at which I am currently attending my care; sadly, they [health professionals] directed me to find medicines in private pharmacies”* [Male, 58, 2 NCDs]

Some patients mentioned that the huge expense of medications affects their relationship with family. *“The problem with my wife is related to expenses, she doesn’t understand the financial burden I am facing to purchase medicines.”* [Male, 45, 2 NCDs]

Health care providers have also described the economic burden of multimorbidity on patients, their family and the government.

*“Patients with multimorbidity are facing a huge financial burden. The expenditure would also extend to affect family and the health system. Health systems suffer depletion of resources because patients with multimorbidity demand more resources.”* [Male 30, Nurse]

The financial burden for some of the patients was related to the ineffective community-based health insurance (CBHI) scheme. Membership to the CBHI does not allow individuals to fully and sustainably access diagnostic and therapeutic services in public hospitals. Some patients come with the anticipation that their CBHI membership helps in getting medicines and laboratory services without difficulty and additional fees. However, neither of these services available consistently in the public hospitals. Hence, patients do not receive the services they need and are eventually forced to either visit private facilities (out of pocket) or return home without receiving the needed services.

*“We pay the premium annually without any interruption, but we are not getting all the services we need. Often times, we do not get laboratory and the choice of medicines here [in the public hospital]. You may get some of the drugs here. If you have money, you may buy the rest outside. If not, you will go with only a few of them and you can imagine what the result will be.”* [Female, 55, 3 NCDs]

Another woman explains about her challenges related to accessing medications*. “We don’t obtain medicines in public facilities. In private pharmacies on the other hand, we are asked to pay 700 to 800 Birr for every regimen; because I don’t have that much money, I keep the prescriptions at home and come for the next appointment hoping to get the drugs for a lower cost in public hospitals.”* [Female, 50, 4 NCDs]

Despite the challenges, senior specialist physicians working both at public and private facilities mentioned that membership to the CBHI scheme is still an important alternative to access most of the essential services.

*“The CBHI is a highly useful mechanism to equip public health facilities and optimize their services; and the cost of health services at public hospital is cheap compared to those in private facilities. I would always recommend people to have a membership to CBHI.””* [Male 50, internist, endocrinologist].

“*Despite some problems with the collection and management of insurance fees, the CBHI is an important avenue for the poor to access chronic NCDs care. Although membership alone may not help getting all of the prescribed medications, patients may get two or three of the medications they need in the facilities integrated with CBHI schemes. It is a useful approach and should rather be strengthened.”* [Male 45, internist, cardiologist]

#### Poor quality of care

Most participants face difficulties in navigating the pathway to chronic outpatient care. The most common challenge in public hospitals is the lack of an easy access to routine medical consultations by physicians. Participants reported that the staff (clerks) working in medical registration rooms are disrespectful, unfair and insulting.

*“The biggest problem I always face is in the ‘card’ [registration] room, the staff working there insult us, and they embarrass and push everyone, even weak patients. The queue is never maintained, they shuffle patient charts and there is a much nepotism.”* [Male, 79, 3 NCDs]

*“….The staff in the ‘card’ room do not listen to you; sometimes they tell you that your chart is lost. Without my children attending with me, I cannot get registered at all. It is by force you keep your turn; otherwise, you will spend the whole day screaming.”* [Female, 50, 3 NCDs]

*“Care provision begins at the gates [with the guards]. The staff working in card room are rude and they do not keep chart orders accordingly. There is a long waiting time; we push each other, no order. I am weak and I could fall down. It is annoying.”* [Male, 74, 3 NCDs]

Participants described that doctors and nurses do not have the time to properly assess and discuss with every patient.

*“Doctors do not offer the opportunity to share concerns and to ask questions. They simply give us a refill prescriptions and rush to do the same for the next patient awaiting. I want my voice to be heard. I want to be checked and reassured. Otherwise, I could get the refills anywhere, may be in pharmacies.”* [Male, 58, 2 NCDs]

In addition, providers do not initiate communication, they do not invite patients to ask questions or share concerns. Some participants feel reluctant to ask questions, because they think providers are busy.

*“I don’t think I have the right to ask the doctors. Doctors appear busy and rushing. I have to accept what they [doctors] say and leave.”* [Female, 50, 2 NCDs]

*“If you appear knowledgeable about to your conditions, they [nurses] embarrass you by saying ‘if you know, don’t come’. Doctors are good.”* [Male, 45, 2 NCDs]

Doctors and nurses have also agreed with the concerns raised by patients. Interviewed providers reported the presence of a large demand (workload) to manage patients with chronic conditions every day.

*“About 30 patients are waiting for me outside. It is unthinkable to give more than five minutes to a given patient, let alone to discuss about their circumstances, needs, priorities and treatment related issues. This is due to the high number of patients we are expected to manage daily.”* [Male 39, Internist].

*“The capacity to pay for and receive multimorbidity care in private facilities often declines gradually. Patients may need to be referred to public facilities where access to a free/subsidized care is somehow available. But there is high workload and problems of integration and optimization of care in public hospitals.”* [Male 45, internist cardiologist]

Patient participants and providers reported that the healthcare system is not designed to foster the most effective support that people with multimorbidity need. People with multimorbidity are often in contact with multiple doctors that are working in different facilities indicating problems of service integration and continuity of care, and the doctors managing patients in public hospitals change often posing additional challenges to the continuity of care.

*“How many times should I tell my personal concerns? Every time I come to this facility, I meet a different doctor. I have to tell him the whole history again. I wish I had one doctor who knows my life and capacities in detail.”* [Male, 50, 2 NCDs]

*“It is true that doctors change every month. Shifting is a norm and we try to record patients’ information on their charts as detail as possible. Patients may have, however, confidential issues that may not be shared with every doctor.”* [Female 37, Nurse]

“*Specialized services in public hospitals are given in scheduled days. For instance Tuesday is for patients with lung diseases; Wednesday for hypertension and heart disease, Thursday for endocrine problems and Friday for renal problems. Therefore, a patient having multiple system diseases should come multiple times in a week, which obviously poses a huge burden for patients and their family.”* [Male 45, internist cardiologist]

Although most patients with chronic NCDs could have comorbid mental illnesses such as depression, physicians generally forget to assess these and refer to appropriate care providers.

*“Associated mental conditions, including depression are often missed. I think doctors lack the awareness that any patient with NCD could have psychiatric problems. Private clinics have no mental health care corners and the referral to other facilities is limited too.”* [Male 45, internist cardiologist]

However, patients attending care in private facilities have the chance to see their physicians regularly and the navigation through the care pathway and support is less challenging compared to public hospitals.

*It is almost 21 years since I began attending diabetic care with Dr…..[Endocrinologist]. They [providers in a private facility] are polite and supportive, they know my diseases, and they teach me about the treatment, diet and follow ups.”* [Female, 60, 2 NCDs]

Lack of patient-centered care was a common problem. Involvement of patients, both in terms of identifying needs and prioritizing interventions to individual context was limited in public facilities.

*“Patient involvement in decision-making is almost none. Patients are too many. Seniors may be consulted to check some patients requiring specialty care. However, specialists have limited time to adequately see every patient we refer. They usually give a refill prescription and there is no opportunity to talk to every patients and identify individual needs. Quality is not a concern for this hospital. The norm is rather to see all patients registered for receiving care on each day.”* [Male 31, GP]

*“Honestly speaking, we have no time to listen to every patient. We have too many patients to manage daily. Doctors have also limited time to address every patient’s concern. Because of the limited time, it is also difficult to teach patients and support them to comply with recommendations.”* [Female 37, Nurse].

*“In private facilities, we try to provide individualized care. Patients are usually involved in decision- making. We empower them. Patient care can be adjusted based on financial capacity, cognition and educational level. However, we do not have guidelines to standardize care for everyone.”* [Male 50, internist endocrinologist]

### Overall capacity and availability of guidelines to manage multimorbidity

Multimorbidity poses a heavy burden on the health system. The volume of work causes difficulties in organizing a formalized system for managing multimorbidity in the practice setting. However, institutions lack the readiness to provide the resources to diagnose and manage multimorbidity.

*“About 90 percent of the patients I manage in public hospital have chronic multimorbidity. They are placing a higher expenditure, consume huge amount of resources and supplies. As a result, public facilities face a lingering stock-out of diagnostic reagents and medicines. The time we take to treat patients with multimorbidity is so long that we often fail to provide individualized and holistic care.”* [Male 45, Internist cardiologist]

Service providers invariably reported that public health facilities are less equipped with diagnostic facilities and medications to manage multimorbidity.

*“Laboratory resources are lacking to identify NCDs and monitor the progress. We are often dependent on physical findings alone.”* [Male 31, GP public hospital]

“*Laboratory service are incomplete, medicines are scarce, even anti-pain [medication]. You feel sad. Patients always complain about these challenges and we have no solutions unfortunately, and the so-called community-based health insurance does not help either. Because most of the diagnostic services covered by CBHI are often unavailable upon request and patients are directed to find them outside [private clinics].” [Female 37, Nurse public hospital]*

*“Most public facilities have limited capacity to manage patient with multiple chronic conditions. Facilities are loaded with high numbers of patients with limited number of resources and staff to effectively manage multimorbidity.” [Male 50, internist, endocrinologist]*

*“Neither diagnostic technologies and reagents nor medicines are sustainably available. There is a weak leadership to plan and procure essential commodities. Those readily available in the market have sub-standard quality. Market inflation and lack of currency [US Dollar] place another challenge to suppliers to provide commodities regularly.”* [Male 45, internist cardiologist]

Providers describe how people with multimorbidity compete for and deplete the scarce resource available to manage other conditions. *“Patients with co-morbidity demand more supplies, more health workers, more infrastructure and consume a disproportionate amount of health care resources and supplies. This will have implication on budget and overall service delivery.”* [Male 30, Nurse]

Lastly, multimorbidity is not addressed in the national treatment guidelines and there is no formalized or standardized system for managing multimorbidity in general practice.

“*I don’t think there is a nationally customized guideline to manage patients with multimorbidity. Doctors do not have standard protocols and the quality of care is sub-optimal in my judgement.”* [Male 30, Nurse]

*“We rely on the science written in text-books and usually, we refer to international treatment guidelines written for an American context. Locally adapted guidelines specify NCDs management in silos. They do not account of the notion of multiple diseases in a given patient.”* [Male 33, internal medicine resident]

In addition, multimorbidity is a hidden problem. Thus, it is not integrated into the health management information system (HMIS).

“*Chronic NCDs are registered and reported individually and the notion of multimorbidity is new for most of us.*” [Female 37, Nurse]

### Perceived satisfaction and quality of care

Most patients attending care in public hospitals were not satisfied with the care they received on the date of interview. The reasons were related to the behavior of staff working in the registration room, long waiting hours, the lack of opportunity to discuss with their doctors, the challenges to obtain drugs and laboratory services, and the lack of education and information, among others.

*“I am not satisfied, because the people working at registration room are rude and the doctors do not offer the chance to ask. They simply write a prescription to find them [drugs] in private. I do not have money. It is disappointing.”* [Male, 58, 2 NCDs]

*“Doctors came late, I am diabetic I want to get the service timely. Poor time management, no medicine.”* [Male, 45, 2 NCDs]

### Suggestions to improve

The majority of the patients suggested improving the problems in the registration (card) room, to sustainably provide medicines and laboratory services in public hospitals, and ensure doctors and other care providers have more time to discuss, educate and provide necessary information. Strengthening CBHI systems is another important area that both patients and care providers underlined.

## Discussion

This study provides a broad description of experiences of individuals living with multimorbidity and perspectives of service providers on multimorbidity and capacity of the health system to screening for and manage multimorbidity.

In general, there was consistency across the individual patient’s stories and the perspectives of service providers on the impact of multimorbidity on patients and the health system.

Findings show that patients with multiple chronic conditions face a wide range of challenges, including difficulty in physical mobility, impaired physical functioning, psychological distress and poor social and community support, and reflect findings from other studies [21, 48].

Patient participants reported suffering from pain and severe physical limitations in doing activities of daily living and performing their organizational and household duties. Such limitations led patients to face both physical and economic dependency. The lack of capacity to earn money leads to difficulties in accessing quality care thus contributing to psychological distress, poor QoL and reduced survival. Our findings are congruent with previous studies [49, 50].

The participants with multimorbidity also reported psychological distress, negative emotional reactions, including sadness due to living with multiple incurable conditions. Some of the psychological disturbances are aspired to the fear of neglect and disrespect by members of the community in their surroundings. The disabling nature of the diseases and the restrictions imposed on some food items and drinks often served in social gatherings made patients to refrain from social engagements. The impact of multimorbidity on psychological and social health has also been reported by other studies [51, 52].

The cost of medicines causes high out of pocket expenses for most patients. The resulting poverty and failure to support their family affected the relationship with their family and the quality of support they receive from them. The lack of social and family support will further compromise patients’ self-management capacity and overwhelm patient resources. These results are similar to those of previous studies [48, 53].

There are complex and interrelated challenges in self-management of multimorbidity as shown in this study. Patients with multimorbidity experience treatment burden related to taking several medications for different diseases. In addition, the possible side effects and drug-to-drug or drug-to-diseases interaction would further impact individuals’ capacity to self-manage and comply with treatment regimens [47, 54]. Some patients in this study reported difficulty experiencing pill burden and having to take multiple medications was the major aspect of treatment burden. Health workers were aware of the inconvenience suffered by patients related to taking several medications. The lack of adherence to treatments would add complexities to the total burden of multimorbidity, QoL and survival [55, 56].

Consistent with previous studies [36, 47], navigating through the chronic NCDs care pathways was difficult for most of the participants, particularly in public facilities. Patients were not adequately supported to move (transit) between care units. There were long waiting times, inequity and mistreatment of patients at entry point to care (registration rooms). Most patients reported experiencing difficulty obtaining their charts on time and they were not treated respectfully and in order of their place in the queue. The lack of a well-organized registration process and communication among service points in public hospitals have contributed to the complexities to receiving timely care and support. Such challenges will eventually compromise the wellbeing and prognosis of individuals with multiple chronic conditions [57].

Participants also reported that the public healthcare system is creating barriers to access the health care they deserve, because of overcrowding, long waiting hours, short consultation time, and lack of counseling, education and information. The lack of access to most diagnostic services and medicines required in public hospitals posed a further major challenge for most patients. Patients reported recurrent stock-out of laboratory reagents and medicines in public facilities, and being forced to find these services in private facilities at high costs. Only patients having the ability to pay for these services in private facilities would have received optimal care, thus denying an equitable health services for the poor, as also reflected in other studies [58].

Service providers described the challenges of managing patients with multimorbidity and inadequacy of health facilities to diagnose and organize care appropriate for this group of patients in a holistic manner, and reflects findings from other studies [59, 60].

The organization of the care for patients with multimorbidity is fragmented, particularly in public facilities. For instance, appointments for different NCDs were arranged on different days, instead of a more convenient arrangement of the different appointments on specific days in consecutive timeslots. Further, given the culture of limited communication on integrated care among doctors, would be consulting different specialists usually in different facilities and receive a variety of, perhaps conflicting, medication or advice [61]. This will further complicate self-management and overall provision of multimorbidity care. Such challenges have also been reported in previous studies [52, 56].

On the other hand, management of patients with co-morbid mental illnesses in private facilities was also fragmented owing to the lack of capacity for in-housing experts in each field of specializations. The presence of untreated co-morbid depression can negatively affect adherence to medications and the lifestyle that are needed to control other medical conditions [24, 62].

Person-centered coordinated care is believed to improve outcomes and experiences of people with multiple-long term conditions [63, 64]. Common factors in this model are regarding the patient as whole person, sharing power and responsibility in decision and establishing personal doctor–patient relationship [65, 66]. However, in public hospitals, the practice of making patients at the center of care provision and decision-making is limited. Most patients described that doctors do not offer the time to ask questions and discuss concerns. Patients’ perception of a lack of compassion from and communication with health care workers, and limited counseling and information provided could lead to poor treatment adherence resulting in complications, including mortality. Some studies have also reported the challenges of providing person-centered care in poor resource settings [67].

How people get access and navigate the care pathway to receive the services they need, the way diagnostic services and medications are made available, the way health services are organized and delivered, and the way in which health care workers communicate with and treat patients are the major quality metrics for people seeking multimorbidity care [21, 32, 47, 68]. However, our finding shows that these quality of care dimensions are not well understood and implemented in the study area.

Consistent with other studies [69], study participants raised several priorities that the health system must address to meeting their needs. The priorities for the participants in this study were: (i) better access to their doctors, medicines and laboratory services, (ii) strengthening CBHI, and (iii) getting enough time to receive counseling, education and information from health care providers.

However, the health system in the study area is not prepared to deal with multimorbidity. It has a constrained capacity to ensure access to diagnostic services and care for treating patients with multiple long-term conditions. Health care providers affirmed that multimorbidity is not well understood and integrated in the health care system in the country, as confirmed by a recent review [6].

### Implications for practice, policy and research

The prevalence of NCDs is rapidly increasing with associated multimorbidity [1, 5, 7]. Multimorbidity affects both patients and the health system through the need for multiple medications, multiple consultations with doctors, and multiple impacts on daily life. The health system must respond to these evolving needs through providing resources and integrated care across service points. Further, living with multimorbidity requires the health system to make available a person-centered approach that improves quality of life and clinical outcomes [65]. Service provision needs to be guided by treatment protocols that also address the possible interaction between physical and mental health proactively from diagnosis to management [70].

Further, it is imperative to understand and address the complex interaction between multimorbidity and socioeconomic deprivation [71]. This includes addressing social determinants of health, financial capacity and optimization of CBHI system to ensure access to essential laboratory services and medicines particularly in public health facilities.

The science of understanding multimorbidity should drive a shift in the way health policies are developed and guide the health care system in tackling this challenge. Policymakers need to better understand how medical education and service configuration should change to meet the needs of people with multimorbidity [70]. Hence, it is clear that priority should be directed to reorient and strengthen the health care services.

It is also imperative to define the best possible model of care for people with multimorbidity. In this sense, the development of treatment guidelines should fuel a reform in the academic curriculum and continuing training programs to accommodate the new scenario in health professions’ education and practice.

In the face of a struggle against communicable and non-communicable diseases, the emergence of multimorbidity is Ethiopia portends a rise in a triple burden of diseases [7]. However, there is a lack of focus on studying the magnitude of multimorbidity, understanding the risk factors associated longitudinally and defining the best model of care. It is imperative to explore the burden of multimorbidity at population level and understand the pattern of disease clustering, its impact on individuals, the society and the healthcare system, and to design the best health care model which is responsive to the current and growing needs of the people with multiple long term conditions, especially those living in poorer socio-economic conditions.

### Strength and limitation of the study

Our findings made a new contribution to our understanding of the burden of multimorbidity on patients and the health system. In addition, we explored the way the health system is organized and its capacity to respond to the complex needs of people with multimorbidity. We got perspectives of both patients and service providers, which maximized the variation in sampling of the study participants However, conducting interview at patients’ homes and inclusion of patient families and staff working at registration rooms, medical laboratory and pharmacy units might have given a broader understanding of the phenomenon under study.

### Conclusion and recommendations

Living with multimorbidity is posing a huge burden on physical, psychological, social and sexual health of patients. Patients seeking multimorbidity care are facing challenges to access care attributable to either financial constrains or the lack of respectful and compassionate health care providers. On the other hand, the health system in the study area is not designed to provide integrated, person-centered and coordinated care for patient living with multimorbidity. Without profound changes to the current views and organization of care, it is highly likely that patients in this category will continue to suffer compromised quality of life, increasing expenditure and premature mortality.

Hence, it is recommended that the health system must understand and respond to the complex care needs of the patients with multimorbidity. There should an enhanced support for patients to get an easy access to the care pathway and the opportunity to talk to their doctors, to play active role in decision making around their care and receive high quality integrated health services. Further, health facilities should devise mechanisms to avail essential resources needed in a sustainable way with reasonable prices. The way in which CBHI is organized could be strengthened to support individual patients and their family in getting equitable access to multimorbidity care. Future research endeavors may need to focus on designing and testing interventions to improve QoL and health service delivery of patients living with multiple long-term conditions in the country.

## Supplementary Information

**Additional file 1.**

## Data Availability

All data generated or analyzed during this study are included in this manuscript.
