# Supplementary material for: Experience of living with multimorbidity and health workers perspectives on the organization of health services for people living with multiple chronic conditions in Bahir Dar, northwest Ethiopia: a qualitative study
Source: BMC Health Serv Res. 2023 Mar 9;23:232. doi: 10.1186/s12913-023-09250-9 (PMC9995260; doi:10.1186/s12913-023-09250-9)
Supplement: Supplementary file 1 — Additional file 1. [file 12913_2023_9250_MOESM1_ESM.zip › S1 (In-depth interview guide patients).docx]

**Multimorbidity of Chronic Non-Communicable Diseases**

**Semi structured Interview Guide for Patient Participants**

My name is………………………………, I am currently engaged in collecting research data for Mr. Fantu Abebe, one of the PhD fellows in Bahir Dar University.

The purpose of the study is to explore how health care services are organized for patients with multiple chronic condition and to understand the perception and lived experiences of patients living with NCDs multimorbidity in Bahir Dar.

**Study procedures**

I will be asking you about socio-demographic characteristics and the types of diseases you are currently living with and your experiences related to health care and living with the health problems you have.

You have the liberty to participate or decline now or any time during data collection. In addition, you have the right to refuse to answer any question or stop the interview at any point or to talk to someone if you fell distressed during the interview. If you choose not to take part in this study, it will not affect the care and support you receive in any way. However, your participation in this study is very important to help us improve our services for people living with long term conditions**.**

**Risk and discomfort: -** The interview may take 30-35 minutes and there is no risk associated with this interview. We will be recording this interview to help us accurately capture the conversation we have with you. Your name will not appear anywhere when we use or report the information.

**Benefits: -** There is little payment to compensate for the time you spend with us.

**Confidentiality**

Any of the information we obtain from you will be kept in strict confidentiality. The information will only be used for the purpose of this study.

**Freedom to ask question or raise concerns**

If you have any question(s) or concern(s) regarding the study, you could ask me or contact the PI using the following contact address.

**Principal investigator:** Fantu Abebe

**II. Semi structured Interview Guide for Qualitative study (Patients)**

**Patient Participants**

| **SN** | **Questions (sociodemographic characteristics)** | **Responses** |  |
| --- | --- | --- | --- |
| 1 | Institution (facility) |  |  |
| 2 | Age |  |  |
| 3 | Sex |  |  |
| 4 | Residence |  |  |
| 5 | Number of conditions a patient is living with |  |  |
| 6 | How long the patient had lived with the chronic conditions for which he/she seeking care at the time data collection? |  |  |

**Themes**

1. Lived experiences of people with NCD multimorbidity
   1. What is it really like to live with several long-term conditions that cannot cured?
2. Daily living (probe: pain, physical limitation, emotion, sleep)
3. In family setting (income, support, relationship)
4. Community setting (probe: social life)
5. Religious institution setting
6. Work setting (status, productivity/performance)
7. Self-management /self-care (medication adherence, appointment, lifestyle behaviors, confidence and locus of control)
8. Experience of care

2.1 How do you describe about the health car and support you receive from the providers?

- 1. Are they taught self-management activities specifically? Were you given guidance on self-management activities? If yes can you tell me about these and if they are working for you and how you manage them?
  2. Health care setting (cost of care, communication with providers, enough time in consultation, addressing needs and preferences, informed decision and point of care)
  3. Do you have challenges in getting healthcare, and when interacting with healthcare professionals and family care givers? (probe, availability of medicines, laboratory service, senior physicians)
  4. How do you describe your satisfaction with care being provided to you?
  5. How could the health and social care systems support you to continue to lead your life?

1. What do you suggest to improve?

Thank you!
