# Supplementary material for: Experience of living with multimorbidity and health workers perspectives on the organization of health services for people living with multiple chronic conditions in Bahir Dar, northwest Ethiopia: a qualitative study
Source: BMC Health Serv Res. 2023 Mar 9;23:232. doi: 10.1186/s12913-023-09250-9 (PMC9995260; doi:10.1186/s12913-023-09250-9)
Supplement: Supplementary file 1 — Additional file 1. [file 12913_2023_9250_MOESM1_ESM.zip › S2 (In-depth interview guide for service providers).docx]

**Multimorbidity of Chronic Non-Communicable Diseases**

**Semi structured Interview Guide for Qualitative study (health service organization and provision)**

**Service providers (Including Medical Directors, Doctors, Matrons, Nurses, Pharmacists and Laboratory Professionals)**

My name is………………………………, I am currently collecting research data for a PhD dissertation in Bahir Dar University.

The purpose of the study is to explore how health care services are organized and being delivered to patients with multiple chronic condition and to understand the perception and practice of service providers on the nature and management of patients living with multimorbidity.

You have the liberty to either participate or decline in this study. In addition, you have the right to refuse to answer any question or stop the interview at any point. However, your participation is very important to help us all understand the nature of health service organization for people living with multiple long term conditions and devise appropriate mechanisms for the same.

**Risk and discomfort: -** The interview may take 30-35 minutes and we will be recording the interview.

**Benefits: -** There is no monetary incentive for participating in this study, however, soft drinks shall be provided.

**Confidentiality**

Your name **will not** be recorded and any of the information we obtain from you will be kept in strict confidentiality.

**Freedom to ask question or raise concerns**

If you have any question(s) or concern(s) regarding the study, you could ask me right now or later by calling using the following contact address.

**Principal investigator:** Fantu Abebe, **Cell phone :**0932-22-5060

**Consent Form**

I understand this information and agree to participate fully under the conditions stated above:

Date: ___________________ Interviewer name: ___________________________________

Thank you!

**I: Service providers (Directors, Doctors, Matrons, Nurses, Pharmacists and Laboratory Professionals)**

**Sociodemographic Characteristics**

| SN | Questions | Responses | Remark |
| --- | --- | --- | --- |
| 1 | Facility |  |  |
| 2 | Profession (responsibility) |  |  |
| 3 | Age |  |  |
| 4 | Sex |  |  |
| 5 | Length of time working in the NCD care context |  |  |

**Themes**

1. Perceived burden of multimorbidity among health care providers (doctors and nurses)
   1. Can you explain what multimorbidity is? (give definition of it if unknown)
   2. Is there an organized information tracking system to identify multimorbidity as an issue? (Probe)
   3. How common is multimorbidity and how much does it affect patients and the care provision?
2. Care of people with NCD multimorbidity (including Lab and pharmacy professionals)
   1. Screening (how people with multimorbidity are identified? who screens?)
   2. Diagnosis (who is making the diagnoses? Sources of data (probe laboratory)
   3. Management: Do these patients need managing in a particular/specific way? (probe, why and how?) (probe, care coordination, team based care, patient involvement in decision making, continuity of care (availability of single point of contact to coordinate all specialist care, including mental health problems), patient education and decision aid (Are there policies, guidelines or protocols that addressed the notion of multimorbidity in place?)
   4. Can you tell us about the principles of patient-centered and integrated chronic care?
   5. Referrals (diagnostic service, prescription, and consultation related)
3. Capacity for managing patients with multimorbidity (Team composition in the care of patient with multimorbidity) (Medical directors, physicians and nurses)
   1. Perceived completeness and continuity of care in place (nurses and doctors)
      1. How complete do you think the care is? Explain; give examples of essential elements
      2. How do you describe the continuity of care for patients with multimorbidity? Explain factors
      3. Sense of capacity, responsibility (ownership) and motivation
   2. Perceived barriers (service related and patient related) (probe, integration, service adequacy, resources, quality; and patient behavior, self-management, coping and follow up)
4. How do you describe about the challenges of multimorbidity?
   - 1. For patients (self-management, attending multiple care points and severity)
     2. For doctors (care coordination, consultation time, managing physical-mental MM)
5. Any other comment:
6. Consideration of interaction between conditions or synergies b/n management of d/t conditions
7. Do you think there is a need to adapt a particular model of care or decision strategy?
8. If yes, what?
9. If no, why?
