# Supplementary material for: Experience of living with multimorbidity and health workers perspectives on the organization of health services for people living with multiple chronic conditions in Bahir Dar, northwest Ethiopia: a qualitative study
Source: BMC Health Serv Res. 2023 Mar 9;23:232. doi: 10.1186/s12913-023-09250-9 (PMC9995260; doi:10.1186/s12913-023-09250-9)
Supplement: Supplementary file 1 — Additional file 1. [file 12913_2023_9250_MOESM1_ESM.zip › S3 (Code book).docx]

Code List

Experiences of Living with Multimorbidity : Bahir Dar Ethiopia

|  |  |  |  |
| --- | --- | --- | --- |
| Questions |  |  |  |
|  | What does living with MM mean to you? |  |  |
|  | Codes | Category | Themes |
|  | No option  Better than dying  Can’t eat what I like  Can’t live as l like  Pain  Fatigue  Lack of sleep  Feeling distressed  Held back  Worried  Stressful  Manage it well  Okay living with it  Unhappy  Can’t adhere to medicine  Feel sad  Distressed  Can’t do anything  Unable to comply with doctors’ advice  Emotional disturbance  Caring myself  Angry  Altered vision  Can’t do my job  Anger  Feeling dependent  Can’t tolerate medications  Depressed  Lack of sleep  Weakness  Altered mobility  Falling  Tired  Can’t move  Pain  Feeling stressed  Pain  Limited mobility  Pain  Limited physical mobility  Anger  Anger  Unable to do routines  Pain  Lack of sleep  Stopped working  Unable to move  Confused  Emotionally disturbed  Limited physical mobility y  Emotional distress  Anger  Anger  Weak  Psychological distress  Feel weak  Limited mobility  Weak feeling  Tiredness  Stay at home  Can’t sleep  Feel weak  Spend a lot to my care  Stopped working  Stopped working at home  Lack of sleep  Distressed  Low libido  Drowsy  Weak  Stopped working my job  Anger  Disappointment  Reduced productivity  Feel dizzy  Restricted to drink what I want  Stress  Lack of focus  Fainting  On dialysis  Limited mobility  Reduced productivity  Lack of sleep  Spend sitting at home  Weak  Intolerant  Fatigue  Stopped working my jobs  Feeling guilty  Stay at home  Lack of sleep  Fear of dying  Feel regret  Restricted to eat what I want  Limited physical mobility  Fear of falling  I use holy water  If I believe I can be cured  Can’t breath  Happy to live till now  Can’t eat what I like  Huge expenditure to medicines  Better that dying  Fatigue  Spend a lot to drugs | Worry  Anger  Distress  Emotional disturbance  Unhappy  Feeling sad  Despair  Depressed  Feeling dependent  Stress  Confused  Guilty feeling  Fear of dying  Feeling rejected  Lack of focus  Pain  Fatigue  Weakness  Stiff joint  Limited physical mobility  Stopped doing jobs  Falling  Lack of sleep  Manage it well  Caring self  Poor adherence  Huge expenditure |  |
|  | Effect of MM on daily living |  |  |
|  | Pain  Discomfort  Pain during movement  Changed food types  Pain  Pain  Altered mobility  Fatigue  Uncomfortable  Pain  Feel weak  Altered vision  Lack of sleep  Pain  Stopped working  Weakness  Unable to climb  Lack of sleep  Weakness  Altered mobility  Falling  Tired  Unable to move  Pain  Distress  Pain  Limited physical mobility  Feeling anger  Feeling anger  Unable to do routines  Pain  Lack of sleep  Stopped working  Stiff joints  Confused  Emotional disturbance  Limited physical mobility  Emotional distress  Feeling anger  Feeling anger  Weakness  Psychological distress  Feeling weakness  Weak  Feeling tired  Sitting at home  Can’t sleep  Feel weak  High expenditure  Stopped working jobs  Stopped working home activities  Lack of sleep  Distress  Reduced sexual drive  Drowsy  Weak  Lack of sleep  Stopped working jobs  Feeling anger  Disappointed  Reduced productivity  Feeling dizzy  Restricted to drink  Stress  Lack of focus  Fainting  On dialysis  Reduced productivity  Limited mobility  Lack of sleep  Spend sitting at home  Weak  Fatigue  Stopped working jobs  Feeling guilty  Stay at home  Lack of sleep  Fear of death |  |  |
|  | Effect of MM on family and their support |  |  |
|  | Happy  Good support from family  Altered sexual function  Unable to lead family  Live alone (widow)  Stopped sex  Poor family support  Unable to get what I need  Conflict with my family  Feeling anger at my wife  Conflict with family  Disagreement  No support from family  Erectile problem  Nagging | Poor psychosocial health  Good family and social support  Sexual problem |  |
|  | Social/community life |  |  |
|  | Enjoy social life  Satisfied  Strong social support  Pressure to choose either drugs or holy water  No social support  No social support  No social support  Stopped social interaction  Stopped social interaction  People don’t listen  People don’t respect  Lip service  Poor social support  Don’t enjoy social life  People don’t understand  Socially isolated  Isolated  Feeling rejected  Stigma  No social support  Limited social activity  Defamation  Stopped social participation  Stopped social participation  No social support  Poor social interaction  No CBHI  Religious practice | Social isolation  Stigma and feeling rejected |  |
|  | No problem  Can’t go to church  Unable to fast  In conflict with myself and values  Unable to fast  Far from church | In conflict with values |  |
|  | Work related |  |  |
|  | Can’t do my routines  Can’t do daily routines  Stopped field works  Spend siting at office  Stopped working  Colleague do my job  Lack of focus  Slow in doing things in office  Changed my job | Impaired functioning/productivity |  |
|  | Self-management |  |  |
|  | Take medications accordingly  Attend follow ups  Stopped working  No fear  Reassure myself  Default medicines during fasting  Skip doses during fasting  Can’t afford for drugs  Expensive medicines  Caring myself  Accepted doctors’ advice  Feel confident managing myself  Unable to comply with dietary modifications  I must take drugs  I should survive  Poor appetite  Changed time of medications intake  Irregularities taking medications | Self-reassured  Good self-management  Poor adherence |  |
|  | 2. Experience of care |  |  |
|  | Health care and support |  |  |
|  | Lack of access to care  Too many patients  Can’t afford shortage of drugs  Dysfunctional dialysis machine  Severe problem in card room  No equity  Long waiting hours  No specialist care  Long waiting hours  Good support from doctors  Queue not maintained  Misplacing orders | Poor access  Long waiting hours  Lack of equity  Poor quality of care |  |
|  | Learning and guidance |  |  |
|  | Less time to talk to doctors  Less time to learn  No opportunity to ask  No teaching  I have to accept what they say only  They tell what I should do  I can’t comment on them  I can’t direct them | Lack of patient centered care  Not empowered |  |
|  | Care provision |  |  |
|  | Can’t get what I need  Can’t ask  Doctors decide  Integrated care  Integrated care  I can’t ask  Obey what they say  Not wise to command them  Lack integration  Lack integration (different appointment days)  Lack of integration  Services in silos  Medication outside  Sabotage getting drugs in public hospitals  Short consultation time  Integrated | Integrated care  Lack of integration  Shortage of drugs  Short consultation time |  |
|  | Access to care, medicines and laboratory services |  |  |
|  | Prescriptions are outside  Expensive medicines  Expensive medicines  Medication outside  Desperation getting drugs  Can’ afford  Can’t obtain medicines in public hospitals  No room to ask  Can’t get medicines  Expensive drugs  Guards are bad  Suffer entering to hospital  Suffer getting drugs  Disappointing  Half lab services  No reimbursement (CBHI)  Long waiting hours  Spend a lot  Rude card room staff  Can’t get drugs  Short consultation time  Drugs outside  Some labs outside  Lack of medications  Expensive in private  Drugs outside  Don’t trust drugs  No order (shuffle patient charts)  Can’t afford private  No specialized services in public  Very expensive medicines  Black market  No prioritized care  Long waiting hours | Shortage of drugs  Expensive drugs  Prescription in private vendors  Difficulty navigating the care pathway  Rude staff  No time to talk to doctors  Poor CBHI scheme management  No fair services |  |
|  | Satisfaction |  |  |
|  | Not satisfied  Good care  Not satisfied  Poor capacity  Not satisfied  Satisfied  Doctors are good  Not satisfied  Don’t talk to you  No physical examination  Simple refill  No need to come and waste time  Long waiting hours | Not satisfied  Poor capacity  Poor quality of care |  |
|  | Support from the care system |  |  |
|  | Don’t give you the time to talk  Short consultation time  No education  Insult  Doctors change every time  Never involved in decision  Poor time management  No discussion  No time to talk | No education |  |
|  | Suggestions to improve |  |  |
|  | Avail services consistently  Improve behavior of staff at card room  Avail medicines  Avail laboratory services  Financial support  Ensure a first-come-first-served  Place sign posts  Clear and readable prescriptions  Care, respect and empathy  Avoid nepotism  Allow enough time to care |  |  |
